# Rational Choice of Reinforcement of Reinforced Concrete Frame Corners Subjected to Opening Bending Moment

**DOI:** 10.3390/ma14123438

**Published:** 2021-06-21

**Authors:** Michał Szczecina, Andrzej Winnicki

**Affiliations:** 1 Faculty of Civil Engineering and Architecture, Kielce University of Technology, Al. Tysiąclecia Państwa Polskiego 7, 25-314 Kielce, Poland; 2 Faculty of Civil Engineering, Cracow University of Technology, Warszawska 24, 31-155 Kraków, Poland; andrzej@hypatia.l5.pk.edu.pl

**Keywords:** concrete, material model, Concrete Damaged Plasticity model, opening bending moment, reinforced concrete frame corners, strut-and-tie method, FEM, Abaqus

## Abstract

This paper discusses a choice of the most rational reinforcement details for frame corners subjected to opening bending moment. Frame corners formed from elements of both the same and different cross section heights are considered. The case of corners formed of elements of different cross section is not considered in Eurocode 2 and is very rarely described in handbooks. Several reinforcement details with both the same and different cross section heights are presented. The authors introduce a new reinforcement detail for the different cross section heights. The considered details are comprised of the primary reinforcement in the form of straight bars and loops and the additional reinforcement in the form of diagonal bars or stirrups or a combination of both diagonal stirrups and bars. Two methods of static analysis, strut-and-tie method (S&T) and finite element method (FEM), are used in the research. FEM calculations are performed with Abaqus software using the Concrete Damaged Plasticity model (CDP) for concrete and the classical metal plasticity model for reinforcing steel. The crucial CDP parameters, relaxation time and dilatation angle, were calibrated in numerical tests in Abaqus. The analysis of results from the S&T and FE methods allowed for the determination of the most rational reinforcement details.

## 1. Introduction

A frame corner under opening bending moment is considered to be a so-called ”D” region in which the distribution of stresses and strains is complicated. While detailing this kind of region, the structural engineer has a few recommendations presented in codes and handbooks and often relies on intuition. The situation is even more complicated in the case of different cross section heights of elements meeting in the corner, as this is not covered in most codes, for example, Eurocode 2 [1]. There are still too few literary studies on the choice of reinforcement in the case of different cross section heights and the article is an attempt to close the gap. Moreover, some of the recommended reinforcement details have a relatively low efficiency factor. A proper choice of the reinforcement of such a region should be based on more sophisticated methods than the handbook recommendations and the authors of this paper suggest using a combination of strut-and-tie and FE methods. This kind of approach has been presented in, for example, the PhD thesis of Akkermann [2], in which a combination of laboratory tests and advanced non-linear FEM calculations was used to analyze reinforced concrete (RC) frame corners under both closing and opening bending moment.

Some researchers [3,4,5,6,7,8,9,10,11,12] performed laboratory tests on RC specimens with various reinforcement details obtaining the corner efficiency factor for specific details. The efficiency factor in the case of laboratory tests can be calculated as (Equation (1)):

(1) $$\eta =\frac{M_{failure}}{M_{capacity}}$$

where *M_failur_*_e_ denotes the opening bending moment causing failure of the corner and *M_capacity_* is a theoretical capacity of an adjacent member, computed as for a RC beam in pure bending. Some chosen results of the laboratory tests of frame corners with different reinforcement details are presented in Table 1. It is worth noting that the laboratory tests were only performed for corners joining elements with the same cross section heights. Example distributions of cracks and failure forms are presented in the works of Johansson [13] and Starosolski [14]. Some recent results concerning reinforced concrete corners are presented in the works of Marzec [15], Wang [16], Berglund and Holström [17], Getachew [18], Haris and Roszevak [19], Abdelwahed [20], and Abdelwahed et al. [21]. They describe laboratory tests, strut-and-tie method approaches, and numerical simulations in various FEM software (e.g., Athena, VecTor2, LS-Dyna).

Eurocode 2 [1] in Appendix J.2.3 gives information only on frame corners joining elements with the same cross section heights. The authors’ aim is to supplement Eurocode 2 (2004) recommendations and investigate the case of the different cross section heights. The authors chose two methods:

(1) Strut-and-tie method (S&T)—to calculate the required reinforcement and to calculate the efficiency factor.
(2) Finite element method (FEM) in Abaqus software using the Concrete Damaged Plasticity (CDP) model for concrete—to calculate the efficiency factor and to recreate the history of loading, the yielding of steel and crack development.

The primary goal of the analysis was to calculate the efficiency factor and crack width for all the details and then to conclude which detail is the most preferable. The secondary, but also important issue of the research was to calibrate some important CDP model parameters. The main motivation to perform all these analyses was to supplement Eurocode 2 [1] and the handbook recommendations with the case of different cross section heights. For this purpose, the authors used the two abovementioned methods and compared the obtained results with the laboratory tests of other researchers (see Section 5).

The reinforcement details taken into considerations are presented in Table 2. Note that details No. 1–7 were used for the cases of both the same and different cross section heights, but details No. 8 and 9 are solely designed for the case of the different cross section heights. Detail No. 1 contains no additional stirrups and diagonal bars, details No. 2 and 3 are two different variations of the type with an additional diagonal bar, and details No. 4–6 are equipped with diagonal stirrups—one or three, those three arranged parallel or “fan-shaped”. The final three details are combinations of diagonal bars and stirrups. Details No. 1–7 and No. 9 are known from handbooks and laboratory tests and detail No. 8 is a new one introduced by the authors.

## 2. Methodology

### 2.1. Strut-and-Tie Method

The S&T method is a modification of a truss analogy (Mörsch [22]). According to Schlaich et al. [23] this modification relies on the application of methods of the theory of plasticity and the redistribution of forces from reinforcing bars to concrete. Compressive stresses are transferred by concrete struts while tensile stresses are transferred by reinforcing bars. The S&T method requires the verification of compressive stresses in struts and nodes. The required reinforcement is calculated from tensile axial forces. Also, it is important to check if compressive stresses in nodes are less than or equal to the ultimate compressive strength. There are three types of S&T nodes. In the CCC node, only compressive struts are connected. In the CCT node, there is also one tensile strut and in the CTT node, there are two tensile struts. The ultimate compressive strength value can be assumed according to different recommendations, e.g., Eurocode 2 [1], Schlaich et al. [23], ACI 318 [24], *fib* Model Code [25]. The authors decided to assume the ultimate compressive stress *σ_Rd,max_* according to Eurocode 2 [1] as follows:

- In struts: *σ_Rd,max_* = *f_cd_*, where *f_cd_* denotes the design compressive strength if a strut is under compression only and 0.6*ν’f_cd_* if a strut is also in tension in a perpendicular direction, where (Equation (2)):
  (2) $$\nu^{\prime }=1-\frac{f_{ck}}{250}$$
- In nodes: CCC node—*σ_Rd,max_* = *ν’f_cd_*, CCT node—*σ_Rd,max_* = 0.85*ν’f_cd_*, CTT node—*σ_Rd,max_* = 0.75*ν’f_cd_*.

The most important issue of the S&T method is the proper choice of a truss model replacing a considered ”D” region. There are various design codes and handbooks that help a designer pick an appropriate scheme, e.g., Schlaich et al. [23], Reineck [26], El-Metwally and Chen [27], but there are few recommendations concerning corners under opening bending moment, see e.g., in Eurocode 2 [1].

### 2.2. Concrete Damaged Plasticity Model for Concrete in FEM Analysis

As mentioned before, FEM calculations were performed with Abaqus software [28] using the Concrete Damaged Plasticity model (CDP) for concrete. There are many other alternative models, e.g., presented in the work Marzec et al. [15] or Cichoń and Winnicki [29,30]. The CDP model for monotonic loading was mathematically formulated by Lubliner et al. [31] and enhanced by Lee and Fenves [32,33] for dynamic and cyclic loading. This model is a combination of the plasticity theory and damage mechanics.

The stress–strain relationship is defined with Equation (3):

(3) $$\sigma =\left(1-d\right)D_{o}^{el}:\left(\varepsilon -\varepsilon_{pl}\right)$$

where *d* is a damage parameter and $D_{o}^{el}$ is an initial stiffness matrix in the elastic state. The damage parameter is given according to Equation (4):

(4) $$1-d=\left(1-s_{t}d_{c}\right)\left(1-s_{c}d_{t}\right)$$

where *d_c_* and *d_t_* are damage parameters and *s_c_* and *s_t_* are stiffness recovery functions in compression and tension, respectively. The yield function in the CDP model is defined according to Equation (5):

(5) $$F=\frac{1}{1-\alpha }\left(\bar{q}-3\alpha \bar{p}+\beta \left(\varepsilon_{pl}\right)\langle {\bar{\hat{\sigma }}}_{\max}\rangle -\gamma \langle -{\bar{\hat{\sigma }}}_{\max}\rangle \right)-{\bar{\sigma }}_{c}\left(\varepsilon_{pl}\right)$$

where *α*, *β* and *γ* are parameters, $\bar{p}$ denotes the effective hydrostatic pressure stress, $\bar{q}$ is von Mises equivalent effective stress and 〈〉 is Macaulay’s bracket. The effective stress is assumed according to Equation (6):

(6) $$\hat{\sigma }=\frac{\sigma }{1-d}$$

The parameter *α* depends on the ratio of the uniaxial compressive strength *f_c_*_0_ to the biaxial compressive strength *f_b_*_0_—see Equation (7):

(7) $$\alpha =\frac{f_{b0}-f_{c0}}{2f_{b0}-f_{c0}}$$

The typical values of the ratio *f_b_*_0_ to *f_c_*_0_ are in the range of 1.10 to 1.16 (Kupfer [34], Lubliner et al. [31]). Parameters *β* and *γ* are calculated as (Equations (8) and (9)):

(8) $$\beta =\frac{3\left(1-K_{T}\right)}{2K_{T}-1}$$

(9) $$\gamma =\frac{3\left(1-K_{C}\right)}{2K_{C}-1}$$

**Figure 1:**
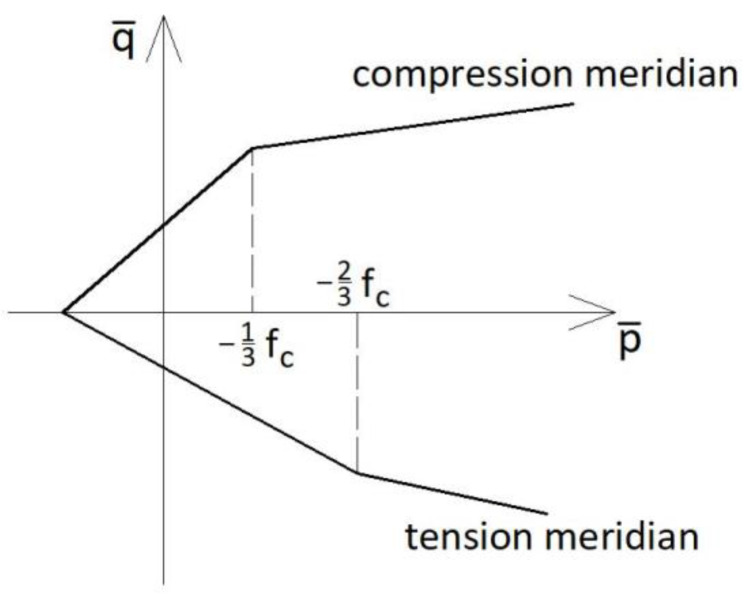
CDP yield surface in the meridian plane.

The parameters *K_T_* and *K_C_* define the shape of the yield surface. Typical values of these parameters vary from 0.56 to 0.61 for *K_T_* and from 0.66 to 0.80 for *K_C_* (Szwed and Kamińska [35]). The yield surface can be presented in the meridian plane—see Figure 1.

**Figure 2:**
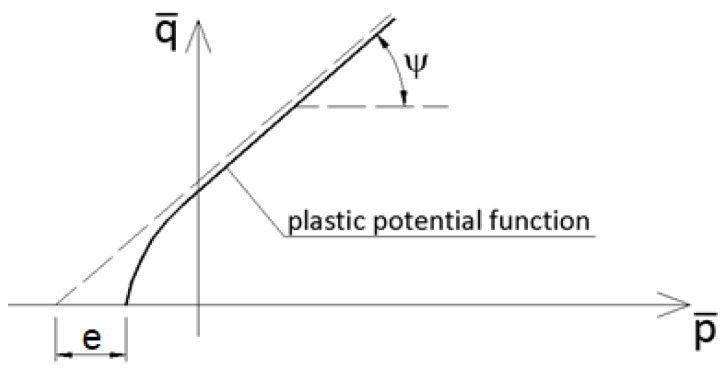
CDP plastic flow potential surface in the meridian plane.

As proposed in [28], the plastic flow potential *G* is assumed as a non-associated potential of the Drucker-Prager hyperbolic type according to Equation (10):

(10) $$G=\sqrt{{\left(e\sigma_{t0}tan\psi \right)}^{2}+{\bar{q}}^{2}}-\bar{p}tan\psi$$

where *e* is an eccentricity parameter and *ψ* denotes a dilatation angle. The graph of the function *G* and a graphical interpretation of *e* and *ψ* are presented in Figure 2.

The viscoplastic regularization can also be applied in the CDP model according to the Duvaut–Lions approach [36] (Equation (11)):

(11) $${\dot{\varepsilon }}_{v}^{pl}=\frac{1}{\mu }\left(\varepsilon^{pl}-\varepsilon_{v}^{pl}\right)$$

where *μ* denotes the relaxation time (the so-called viscosity parameter in Abaqus code) and the bottom index *ν* refers to the viscous part of plastic strains. The viscoplastic behavior of concrete is taken into consideration in the CDP model only if the relaxation time is larger than zero.

**Figure 3:**
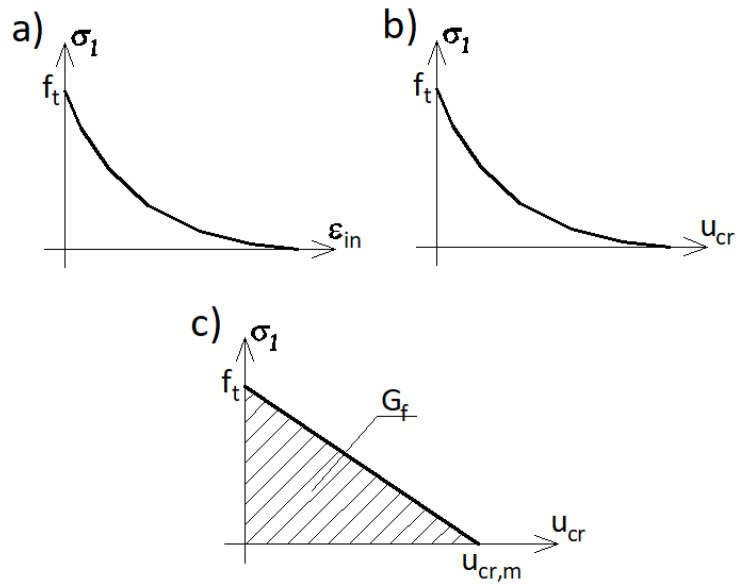
(**a**–**c**) CDP plastic flow potential surface in the meridian plane.

A full definition of the CDP model needs a specification of a few parameters, namely:

(1) The stress–strain relationship defining a compressive behavior of concrete, usually in a form of a set of points;
(2) The dilatation angle *ψ* in the $\bar{p}-\bar{q}$ plane;
(3) The flow potential eccentricity *e*;
(4) The ratio *f*_*b*0_/*f*_*c*0_ of the biaxial compressive strength to the uniaxial compressive strength;
(5) The ratio *K* of the second stress invariant on the tensile meridian to that on the compressive meridian for the yield function;
(6) The tension behavior of concrete in the post-critical range in Abaqus can be defined in three different ways (see Figure 3), namely, as coordinates of points on *σ–ε_in_* curve in a tabular form called STRA in Abaqus code (Figure 3a), *σ–u_cr_* curve called DISP (Figure 3b), or the fracture energy *G_f_* called GFTEN (Figure 3c).

The option GFTEN is equivalent to the option DISP with a linear *σ–u_cr_* relation and *u_cr,m_* = 2*G_f_/f_t_*. Because the model formulation is a continuous one and defined in terms of the stress–strain relation rather than stress–displacement, in the numerical implementation the options DISP and GFTEN are transformed to the *σ–ε_in_* relation depending on the size of the given finite element based on the so-called crack band approach (Bažant and Oh [37]).

There are also two optional CDP model parameters: the relaxation time and the damage conditions, the latter defined separately for compression and tension.

The proper choice of the dilatation angle and relaxation time is still an open scientific issue. The proper values of both parameters are crucial for obtaining reasonable results from FEM computations (Szczecina and Winnicki [38]). Different values of the dilatation angle for concrete were suggested by the researchers listed in Table 3. The role of the dilatation angle in plasticity-based models was discussed in depth in a paper by Wosatko et al. [39]. The range of the proposed values is very wide, and this is why the authors decided to perform a calibration and validation of the dilatation angle for concrete. A similar situation occurs for the relaxation time—some researchers (e.g., Genikomsou and Polak [40,41] and Pereira et al. [42]) proposed values from 10^−5^ s to 10^−4^ s. The calibration and validation of the dilatation angle and relaxation time are described in the next section.

## 3. Calibration and Validation of CDP Model Parameters

### 3.1. Uniaxial and Biaxial Compression Tests

**Figure 4:**
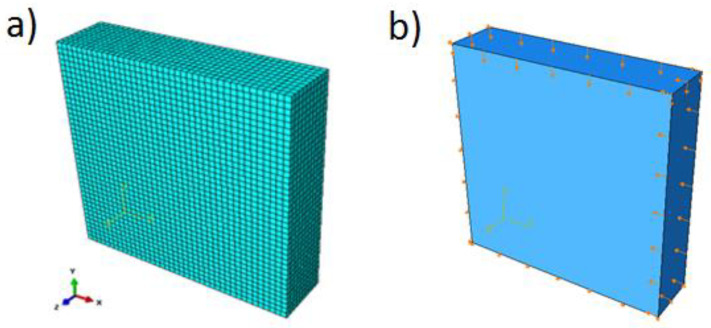
Setup for compression test: mesh (**a**) and boundary conditions (**b**).

The dilatation angle was calibrated in uniaxial and biaxial tests performed numerically with Abaqus code. The geometry and boundary conditions of the specimen under compression are shown in Figure 4. The loading regime is defined as uniaxial or biaxial compression. The stress–strain curve in uniaxial compression was calibrated to match the experimental results of Kupfer [34].

**Figure 5:**
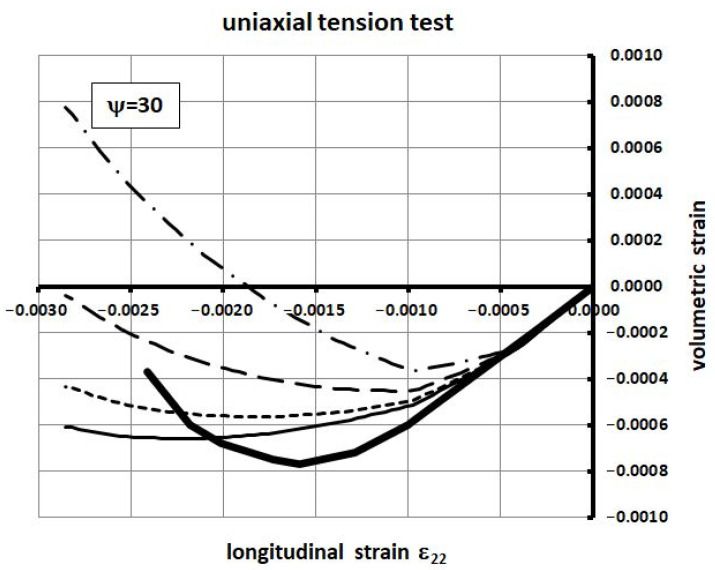
Volumetric strain vs. longitudinal strain for different dilatation angle values in the uniaxial compression test.

**Figure 6:**
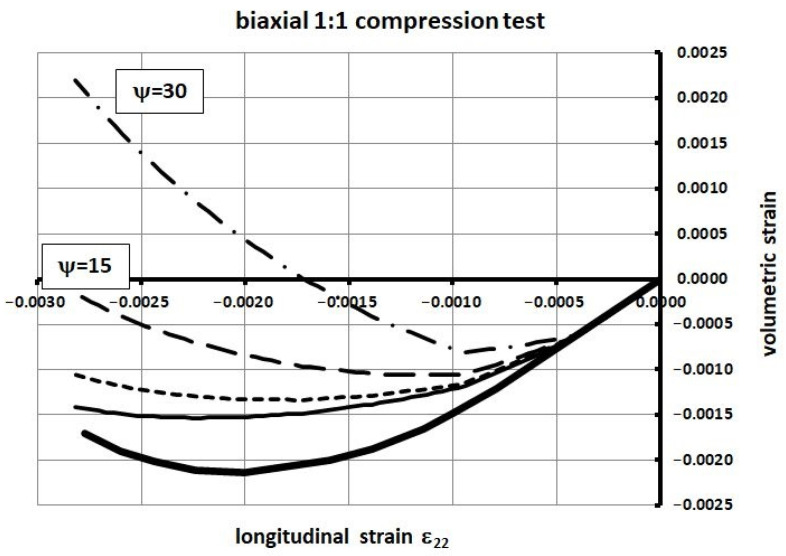
Volumetric strain vs. longitudinal strain for different dilatation angle values in the biaxial 1:1 compression test.

The material properties of concrete are as follows: compressive strength *f_c_* = 34.30 MPa; tensile strength *f_t_* = 3.5 MPa; elastic modulus *E_c_* = 35 GPa; Poisson’s ratio *ν_c_* = 0.167; fracture energy *G_f_* = 146.5 N/m. Four values of the dilatation angle were tested, namely 0, 5, 15, and 30 degrees. The relationship between the volumetric strain *ε_v_* and linear strain *ε*_11_ is shown in Figure 5 and Figure 6. For relatively small values of the dilatation angle (0 to 15 degrees) the volumetric strains obtained in numerical computations were similar to those of Kupfer [34]. In the laboratory tests the volumetric strains were negative, which means the compaction of concrete. In the numerical simulations they remained negative when the dilatation angle was in the range of 0 to 15 degrees. In the case of 30 degrees, large positive volumetric strains in the post-critical range occurred. For that reason, the authors suggest using relatively low values of the dilatation angle **in the range of 0 to 15 degrees**. If the higher values are used the stiffness and bearing capacity of concrete elements can be overestimated in the case of confinement, e.g., in the plane strain case.

### 3.2. Uniaxial Tension Test

**Figure 7:**
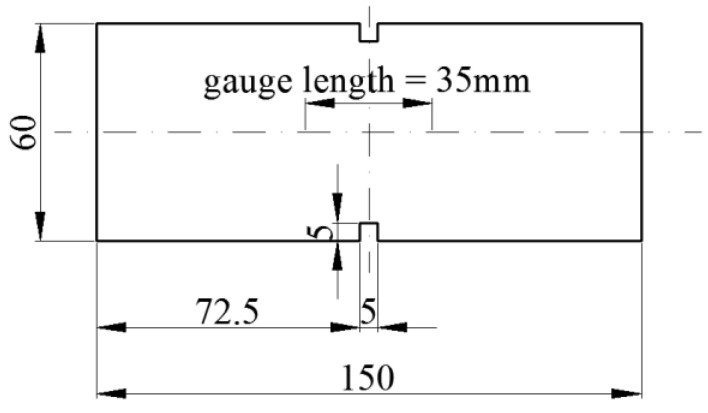
Geometry of specimen in the uniaxial tension test; dimensions in (mm).

**Figure 8:**
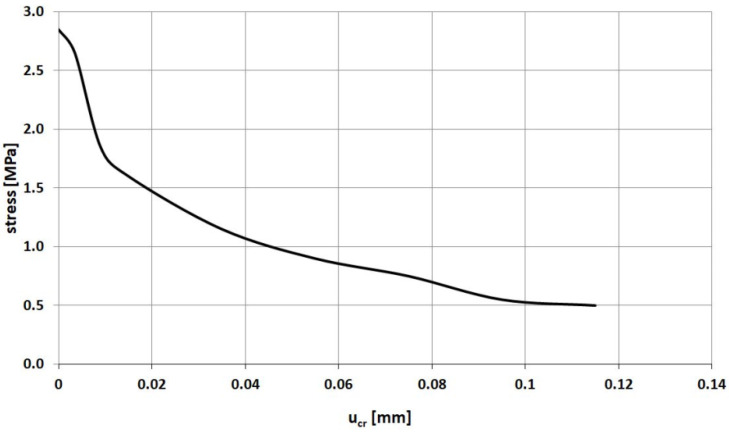
Crack width vs. stress relationship assumed in the uniaxial tension test.

The geometry of a specimen under tension is presented in Figure 7. Two-dimensional elements in the plane stress state were used in the FE model of the specimen. The notches force crack localization in the middle cross section of the specimen. The properties and geometry of the specimen were taken from Woliński’s research [52]. A displacement is imposed at the right edge and the left edge is fixed. The material properties of concrete are as follows: compressive strength *f_c_* = 34.30 MPa; tensile strength *f_t_* = 3.5 MPa; elastic modulus *E_c_* = 35 GPa; Poisson’s ratio *ν_c_* = 0.167. The tensile behavior of concrete is defined as a set of points on the *σ–u_cr_* curve taken from Woliński’s research—see Figure 8.

**Figure 9:**
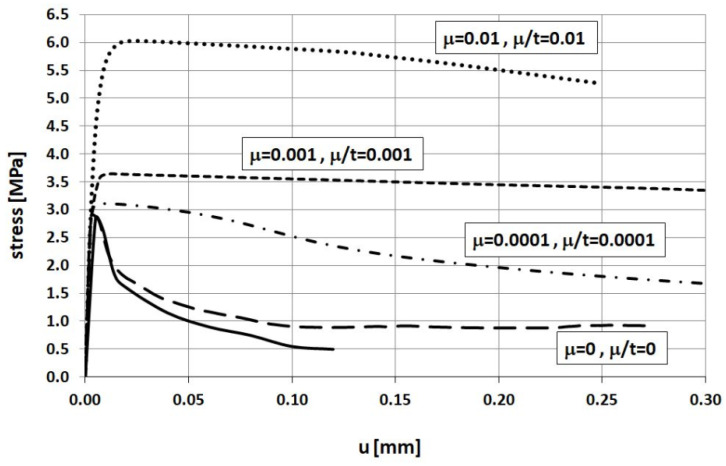
Displacement vs. stress relationship obtained for different relaxation time values.

**Figure 10:**
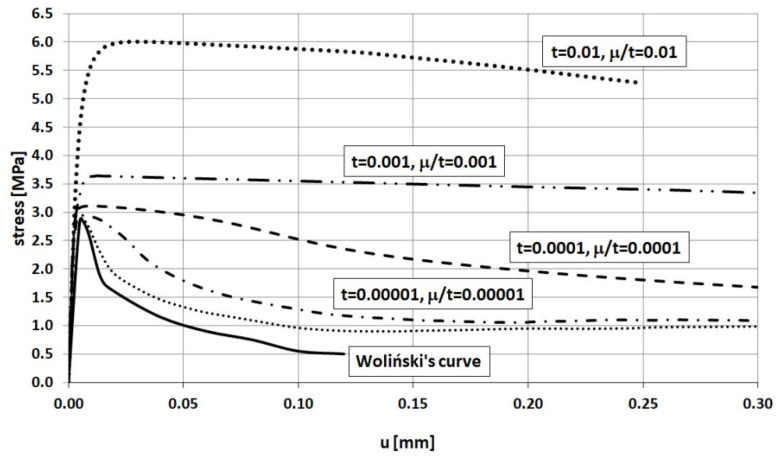
Displacement vs. stress relationship obtained for different relaxation time values.

The relationship between stress and displacement obtained in FEM analysis for various relaxation time values is presented in Figure 9 and compared with Woliński’s experimental curve. These results are obtained for different values of the relaxation time *μ*, namely 0, 0.0001, 0.001, and 0.01 s keeping a fixed loading time value of 1 s. In turn, Figure 10 shows the results of the same experiment for different values of the loading time *t* keeping a fixed relaxation time value of 0.0001 s. A comparison of Figure 9 and Figure 10 demonstrates that the same results are obtained for the same values of the ratio *μ/t*. Therefore, it can be concluded that a key factor in viscoplastic regularization **is not the value** of the relaxation time itself but the ratio *μ/t*. In order to obtain results that are close enough to reality the authors recommend using **a value of *μ/t* equal to 0.0001 or less**.

**Figure 11:**
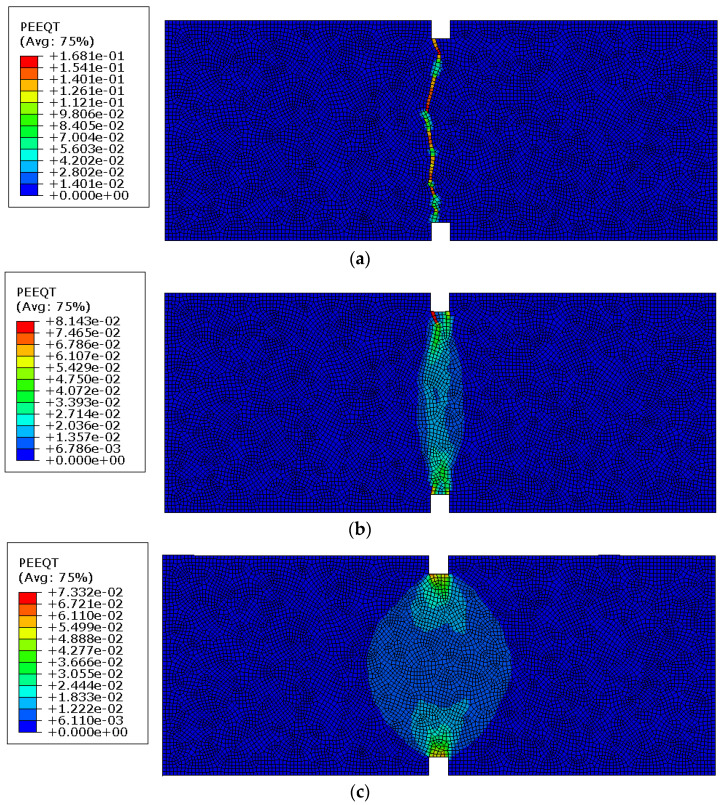
(**a**) Crack pattern, relaxation time equal to 0. (**b**) Crack pattern, relaxation time equal to 0.0001 s. (**c**) Crack pattern, relaxation time equal to 0.001 s.

Figure 11 presents crack patterns (i.e., values of the plastic equivalent strains in tension PEEQT in Abaqus code) for the numerical analyses with a fixed value of loading time equal to 1 s and different relaxation times (the viscosity parameter) equal to 0, 0.0001, and 0.001 s leading to the ratios *μ/t* of 0, 0.0001, and 0.001, respectively, using a FE mesh size of 1 mm (Szczecina and Winnicki [53]). The stress–displacement curves pertinent to these numerical analyses are shown in Figure 9.

For the zero value of the viscosity parameter when the regularization is not taken into account, a distinct crack along the whole cross section in the notched area occurs. As the crack is localized in one row of finite elements, the width of the damaged region clearly depends on FE discretization assumed in the model. A clear damage location is indicated by the approximately vertical crack. The crack and damage zone change with the growth of the viscosity parameter. A constant damage zone (around 5 mm width) can be observed. Larger values of the parameter can cause the spread of the damaged zone outside the notched cross section. This observation confirms the authors’ opinion that the ratio *μ/t* should not exceed a value of 0.0001.

## 4. Strut-and-Tie and FEM Analysis of Corners under Opening Bending Moment

**Figure 12:**
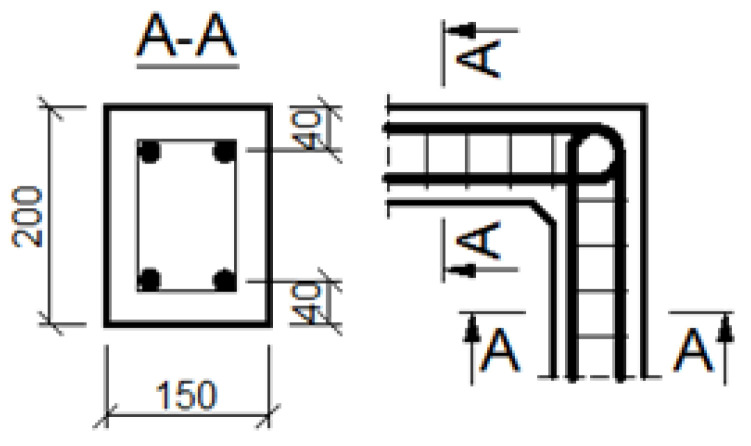
Geometry and main reinforcement for the case of elements with the same cross section heights; dimensions in (mm).

**Figure 13:**
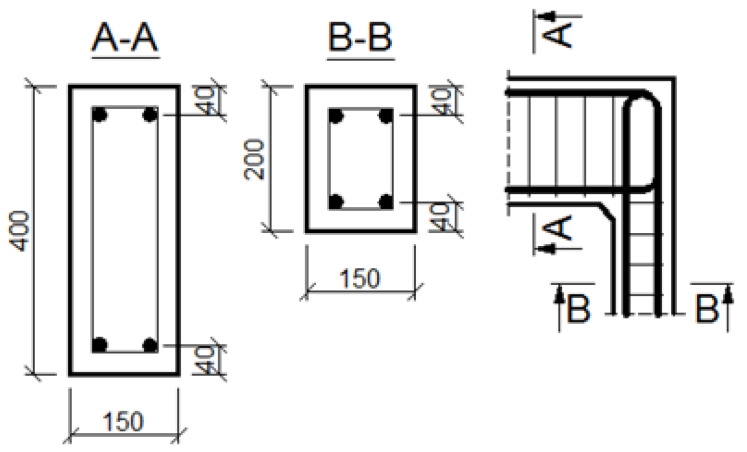
Geometry and main reinforcement for the case of elements with different cross section heights; dimension in (mm).

The general geometry and main reinforcement of the corners are presented in Figure 12 and Figure 13, where dimensions are given in (mm). Two different cases are taken into account: when cross section heights of elements joining in the corner are the same and when they are different, where the properties of the specimen in the first case are comparable to those applied by Mayfield et al. [4]. The analyzed corners have the same common properties, which are listed below. All the corners are made of concrete C50/60 and reinforced with steel B500SP. Material constants are taken as the design values according to Eurocode 2 [1]:

- Concrete: *f_c_* = 34.30 MPa, *E_c_* = 35 GPa, *ν* = 0.167, *f_t_* = 3.5 MPa, *G_f_* = 146.5 N/m;
- Reinforcing steel: *f_y_* = 434.8 MPa, *E_s_* = 200 GPa, *ν* = 0.3.

**Figure 14:**
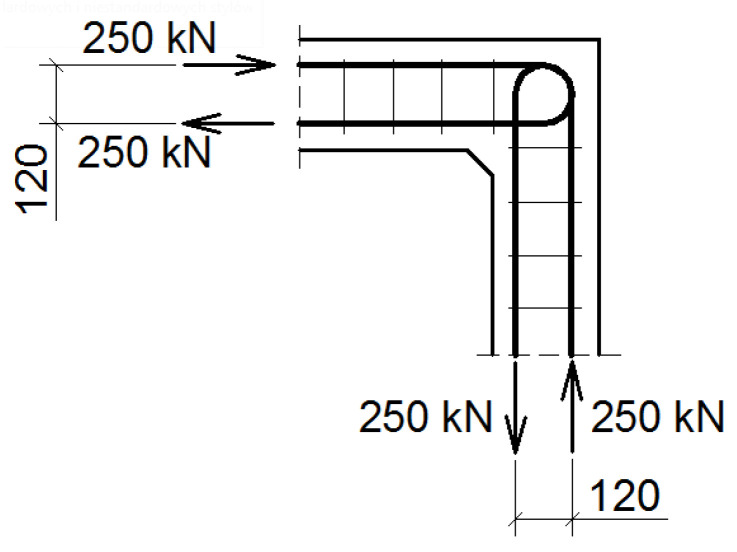
Geometry and main reinforcement for the case of elements with different cross section heights; dimensions in (mm).

**Figure 15:**
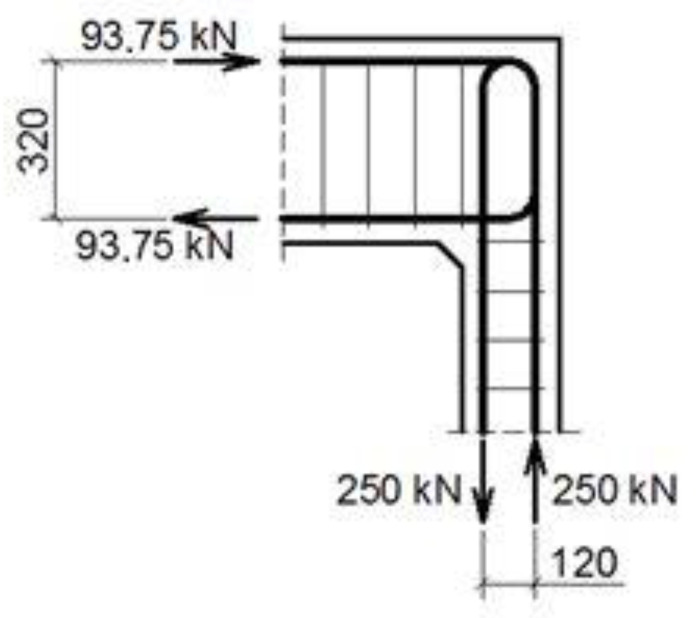
Loading scheme for the case of elements with different cross section heights; dimensions in (mm).

Each corner is subjected to an opening bending moment with a reference value of *M_ref_* = 30 kNm modeled as a pair of forces—see Figure 14 and Figure 15. Seven different reinforcement details taken into consideration are listed in Table 2. In this section, corners are subjected to a pure bending moment, but in the Section 5.2. there is an example of a calculation where the moment is accompanied with normal and shear forces, which is a very important case in practice.

### 4.1. Calculations in the Strut-and-Tie Method

#### 4.1.1. The Case of Elements with the Same Cross Section Heights

**Figure 16:**
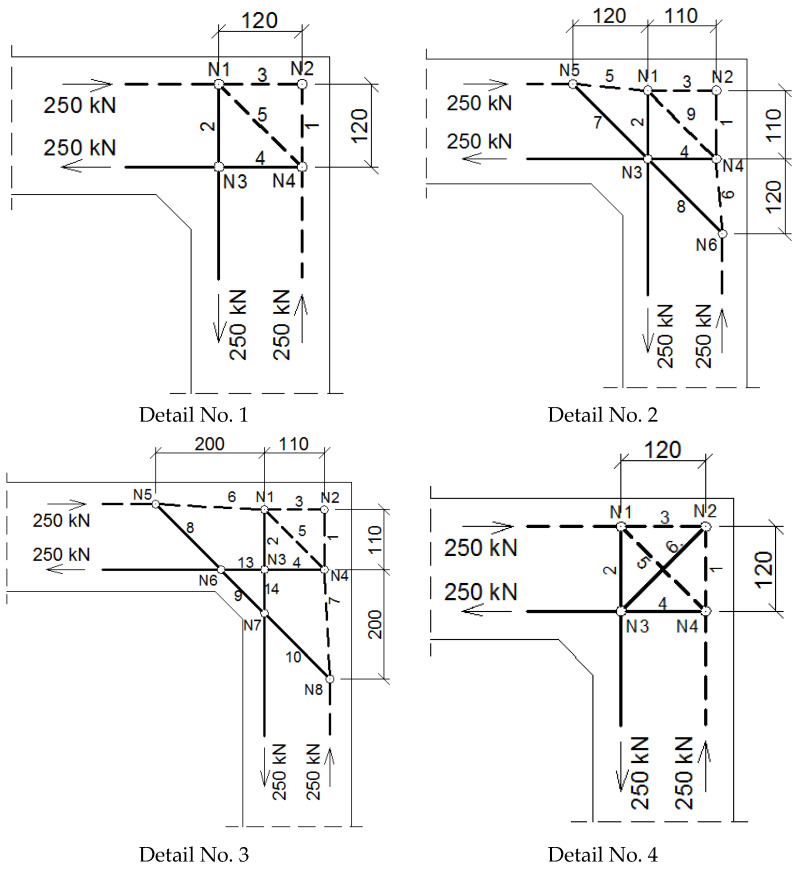

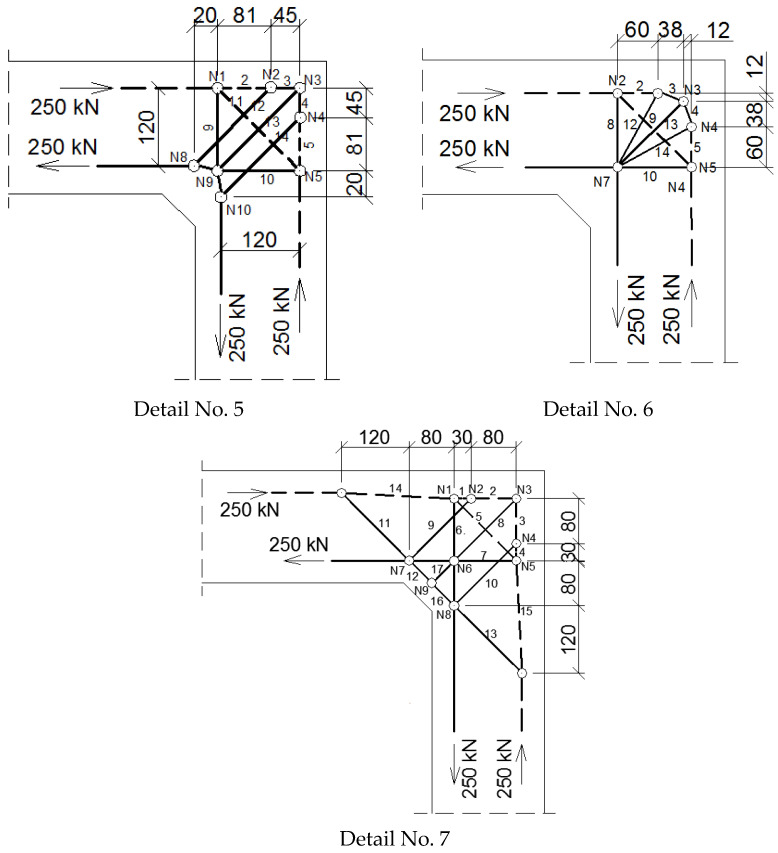
Truss schemes assumed for each reinforcement detail in the S&T method; dimensions in (mm).

The assumed truss schemes for each reinforcement detail are presented in Figure 16. It is appropriate to stress that the presented schemes have been devised and developed by the authors of this paper rather than merely being adapted from schemes taken from the literature. An S&T analysis was performed for each corner detail using the reference value of the bending moment *M_ref_* = 30 kNm. The main reinforcement of the elements joined in the corner was chosen as a pair of 2ϕ20, top and bottom (the reinforcement ratio calculated for the 200-mm-high elements joined in the corner equal to 0.0262). For the main reinforcement, the carrying capacity of the RC cross section was computed according to Eurocode 2 [1] (for cross section dimensions—see Figure 12, section A-A and Figure 14, section B-B) leading to *M_capacity_* = 32.7 kNm and the ratio (Equation (12)):

(12) $$\omega =\frac{M_{capacity}}{M_{ref}}=\frac{32.7\mathrm{kNm}}{30.0\mathrm{kNm}}=1.09$$

The corner efficiency ratio was computed using Equation (1), leading to Equation (13):

(13) $$\eta =\frac{M_{failure}}{M_{capacity}}=\frac{M_{failure}}{M_{ref}}\cdot \frac{M_{ref}}{M_{capacity}}=\frac{M_{failure}}{M_{ref}}\cdot \frac{1}{\omega }$$

For any strut, tie, or node of the given S&T scheme a linear relation according to Equation (14) holds:

(14) $$\sigma_{i}=a_{i}\cdot M_{ref}$$

where *a_i_* is the proportionality factor and the subscript *i* denotes a number of the S&T scheme element (*i* = 1, 2, 3 … *N*, where *N* is a number of the elements). Because, by definition, the S&T method is linear, for the bending moment at the point of failure the same relation is valid (Equation (15)):

(15) $$\sigma_{Rd,max,i}=a_{i}\cdot M_{failure}$$

where, this time, *i* is the number of the weakest strut/tie/node at the corner. Combining Equations (14) and (15), Equation (16) is obtained:

(16) $$\frac{M_{failure}}{M_{ref}}=\underset{i=1\ldots N}{min}\left(\frac{\sigma_{Rd,max,i}}{\sigma_{i}}\right)$$

In Equation (16), the operator “*min*” is used to find the weakest strut/tie/node *i* in the whole set *i* = 1, 2, 3 … *N*. Inserting Equation (16) in Equation (13), a formula is eventually found which enables one to compute the corner efficiency ratio using the stress values *σ_i_* obtained in the S&T analysis for *M_ref_* = 30 kNm (Equation (17)):

(17) $$\eta =\underset{i=1\ldots N}{\min}\left(\frac{\sigma_{Rd,\max,i}}{\sigma_{i}}\right)\cdot \frac{1}{\omega }$$

In Table 4, the corner efficiency factors obtained with the S&T method for all the reinforcement details are presented. The efficiency factor was calculated according to Formula (17). For all the details and all the cross-section heights the required reinforcement is calculated, and then the proper reinforcement is provided in FEM models in Abaqus. The results listed in Table 4 show that the use of at least one diagonal stirrup allows for obtaining relatively high efficiency factors. If there are no diagonal stirrups in the corner (detail No. 1) or only a diagonal bar is provided (details No. 2 and 3), no significant improvement to the efficiency factor is seen. The highest efficiency factor is obtained using reinforcement detail No. 7.

#### 4.1.2. The Case of Elements with Different Cross Section Heights

**Figure 17:**
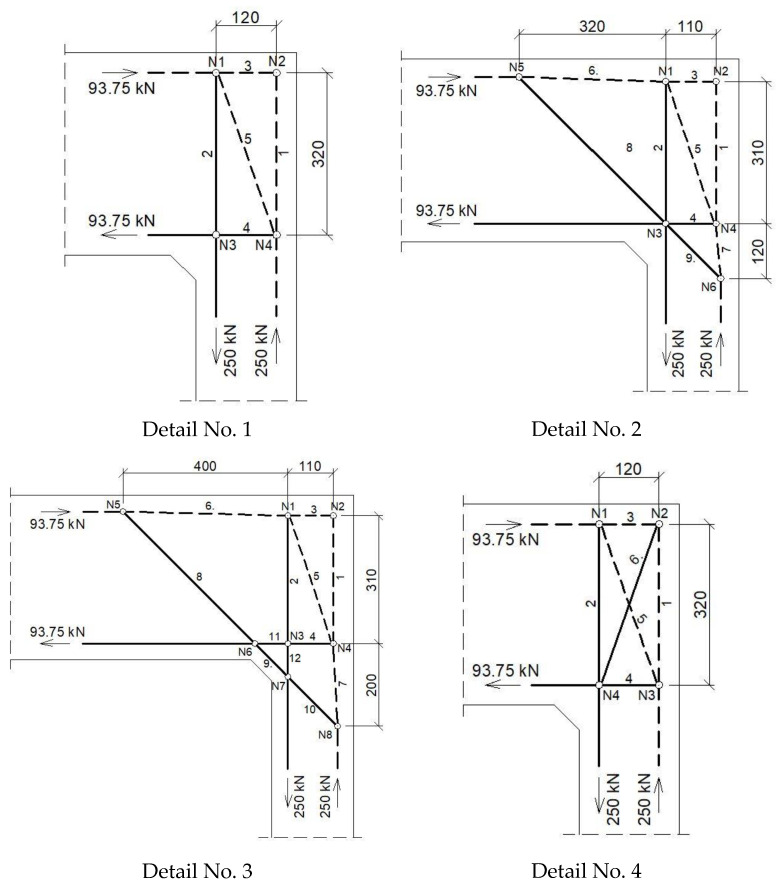

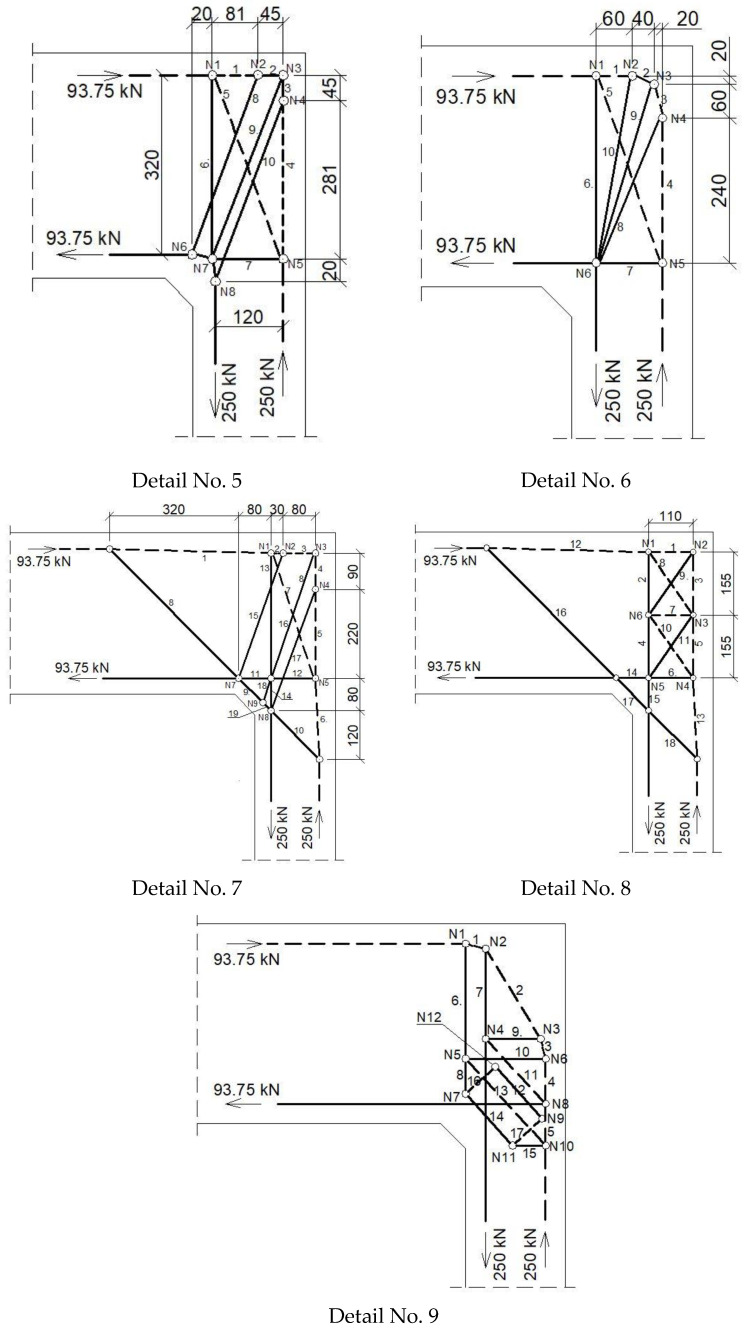
Truss schemes assumed for each reinforcement detail in the S&T method; dimensions in (mm).

The authors’ truss schemes for this case are presented in Figure 17. Please note that this time, nine different reinforcement details were taken into consideration. Seven of these are comparable to those analyzed for the case of corners with elements of the same cross section heights and the two last details are only for the case of elements with different cross section heights. The efficiency factors and provided reinforcement are presented in Table 5. Some similar conclusions can be drawn as those drawn in the case of elements with the same cross section heights. The use of at least one diagonal stirrup causes a significant growth of the efficiency factor, wherein the best result is obtained for detail No. 4. However, the efficiency factor for detail No. 9 is lower than 1 and so far, only the details from No. 4 to 8 can be recommended for practical use.

### 4.2. Calculations in FEM

#### 4.2.1. The Case of Elements with the Same Cross Section Heights

**Figure 18:**
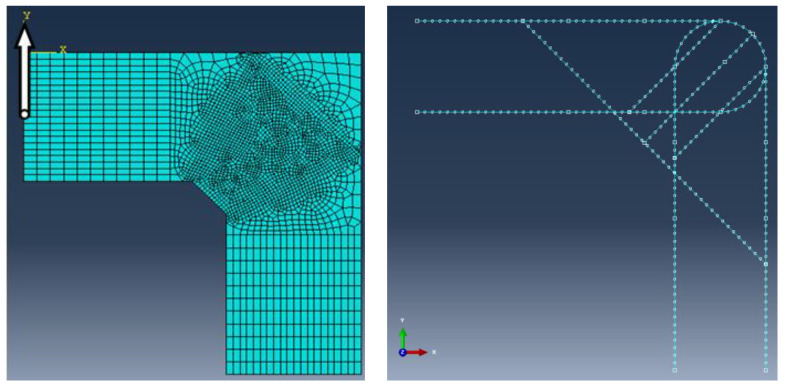
Meshing of concrete and reinforcing steel (detail NO. 7) and analyzed nodal displacement.

**Figure 19:**
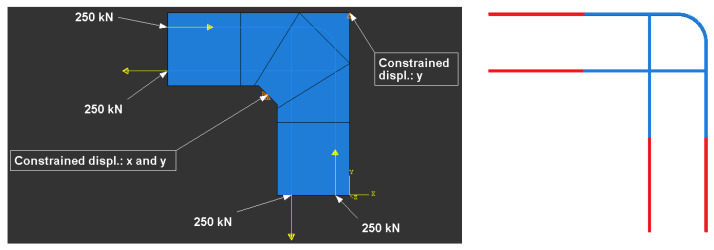
Boundary conditions and ideally elastic fragments of reinforcement (in red).

FEM calculations were performed using Abaqus/CAE ver. 6-12.2 [28]. The meshing of the concrete specimen and reinforcing steel is presented in Figure 18, where the meshing of the reinforcing steel is pertinent to reinforcement detail No. 7. The boundary conditions and loading of the corner are presented in Figure 19. To avoid the localization of strains and stresses and numerical problems connected with the concentrated forces acting on the specimen, some fragments of the main reinforcement and concrete outside the corner zone were defined as ideally elastic. A range of the elastic fragments is shown in Figure 19. Full bond was assumed between concrete and reinforcing steel using the “embedded region” option in Abaqus code.

Each corner was analyzed under the reference opening bending moment *M_ref_* = 30 kNm modeled as a pair of forces. The reinforcement was first calculated with the S&T method and then put in the FEM model. The corners are calculated in both the plane stress and the plane strain states. The applied load was defined with a load parameter *λ* whose value of 1 represents the reference value of the opening bending moment *M_ref_* = 30 kNm. As presented in Section 4.1 the carrying capacity value of the bending moment is *M_capacity_* = 32.7 kNm. The FE analysis, on the other hand, is performed in a similar manner to the S&T analysis using the reference value of the bending moment *M_ref_*. Therefore, the load parameter is defined as (Equation (18)):

(18) $$\lambda =\frac{M}{M_{ref}}$$

Especially for the failure holds (Equation (19)):

(19) $$\lambda_{failure}=\frac{M_{failure}}{M_{ref}}=\frac{M_{failure}}{M_{capacity}}\cdot \frac{M_{capacity}}{M_{ref}}=\eta \omega$$

Thus, the value of the efficiency factor *η* = 1.0 corresponds to the load parameter $\lambda_{failure}$ (Equation (20)):

(20) $$\lambda_{failure}=1.0\omega =1.09$$

For the purpose of material behavior modeling in Abaqus software, the CDP model for concrete and the classical metal plasticity model for steel were assumed. The stress–strain curve for concrete in uniaxial compression was approximated with the *σ–ε* curve according to Eurocode 2 [1]. For concrete behavior in uniaxial tension the fracture energy *G_f_* option was set as input in Abaqus. The used values of the CDP parameters are listed in Table 6.

A load time of 1 s and the ratio *μ/t* = 0.0001 were assumed. The main results obtained directly from FEM calculations are the equivalent plastic strains in tension PEEQT and the damage parameter in tension DAMAGET for concrete and von Mises’ stress and the yield flag for reinforcing steel. The relationship between the chosen nodal displacement and the load parameter *λ* is plotted as a graph. Moreover, crack width is calculated using values of PEEQT and the formula given by Červenka et al. [54] (Equation (21)):

(21) $$w=\varepsilon_{cr}\cdot \left(1+\left(\gamma_{\max}-1\right)\cdot \frac{\theta }{45}\right)\cdot L_{t}$$

where *L_t_* is a finite element width perpendicular to the direction of the crack, *ε_cr_* denotes tensile strain in the cracked element, *γ_max_* is assumed as equal to 1.5, and *θ* is the crack propagation angle (given in degrees).

**Figure 20:**
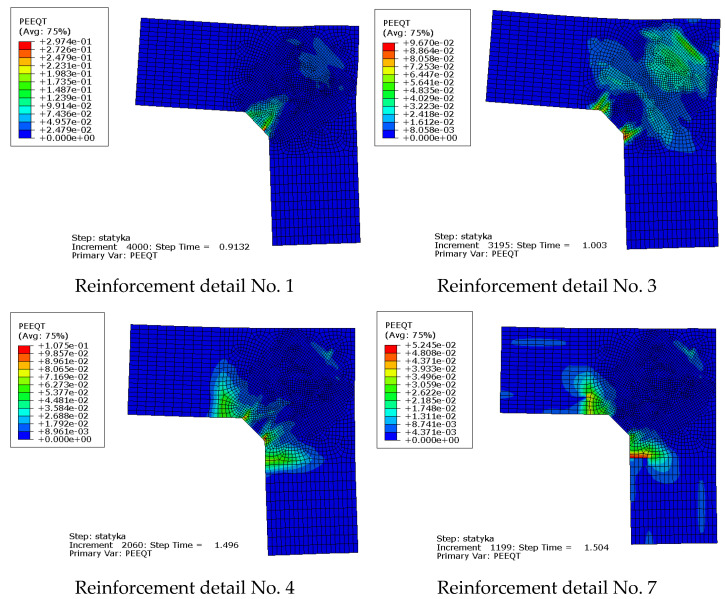
Equivalent plastic strains in tension PEEQT for chosen reinforcement details in the plane stress state.

**Figure 21:**
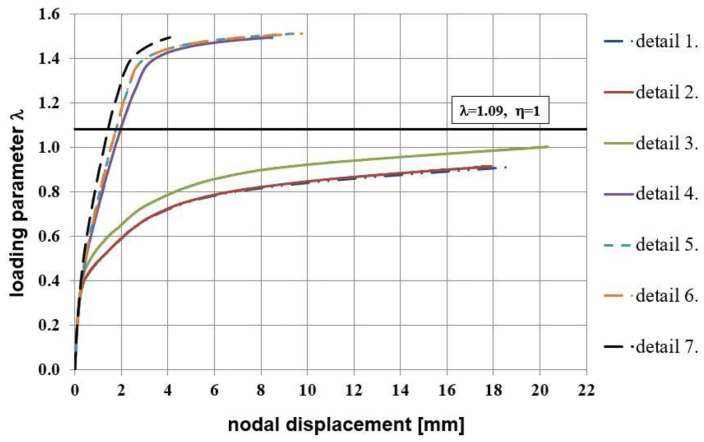
Nodal displacement vs. loading parameter in the plane stress state.

Maps of the PEEQT for some chosen reinforcement details in the plane stress state are presented in Figure 20. For the sake of brevity only details No. 1, 3, 4, and 7 are discussed. The use of at least one diagonal stirrup causes cracks to occur outside the corner zone, which is clearly visible. A relationship between the nodal displacement and loading parameter is presented in Figure 21.

**Figure 22:**
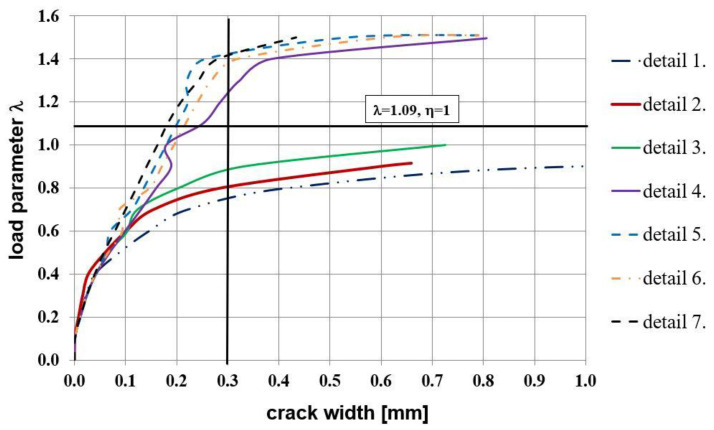
Crack width vs. loading parameter in the plane stress state.

Analyzing the relationship presented in Figure 21 the stiffer behavior of corners equipped with reinforcement details No. 4 to 7 is seen. Finally, the relationship of crack width versus loading parameter presented in Figure 22 once more confirms that the use of diagonal stirrups either alone or combined with diagonal bars is recommendable for corners under opening bending moment. In this graph a commonly assumed value of ultimate crack width, namely 0.3 mm [1], is marked with a vertical line. It is clearly seen that for reinforcement details No. 4 to 7 this ultimate value is reached for relatively high loading parameter values.

Values of the efficiency factors for all the details and both methods are listed in Table 7. Higher values of this factor for reinforcement details No. 4 to 7 are seen, confirming that these details are recommendable for practical use. Moreover, the results obtained with FEM in the plane stress state are comparable with those obtained with the S&T method. However, it should be remembered that a full analysis of a corner under opening bending moment demands not only the calculation of the efficiency factor but also checking the crack width.

To summarize this subsection the authors state that the use of at least one diagonal stirrup significantly helps to limit crack propagation and crack width in the corner zone and to reach relatively high values of the corner efficiency factor in the case of elements with the same cross section heights. The best results seem to occur for reinforcement detail No. 7. By contrast, the use of a diagonal bar without diagonal stirrups (details No. 2 and 3) does not help at all.

#### 4.2.2. The Case of Elements with Different Cross Section Heights

**Figure 23:**
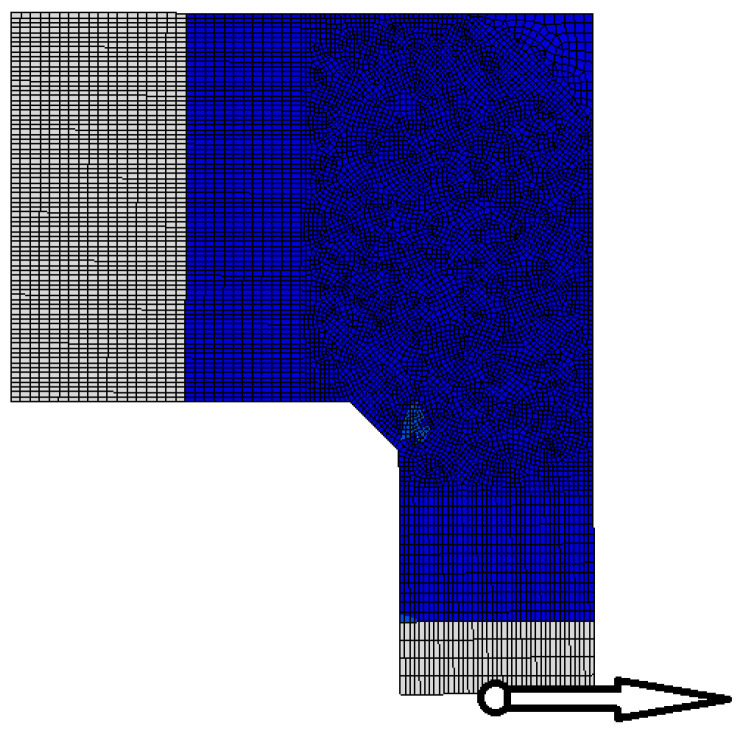
Meshing of frame corner and analyzed nodal displacement for the case of elements with different cross section heights.

In this case in FEM analysis, the only difference in comparison with the previous case is the geometry of the concrete specimen and reinforcement, and the locus of a nodal displacement—see Figure 23. The rest of the input data, including the value of bending moment, remains unchanged.

**Figure 24:**
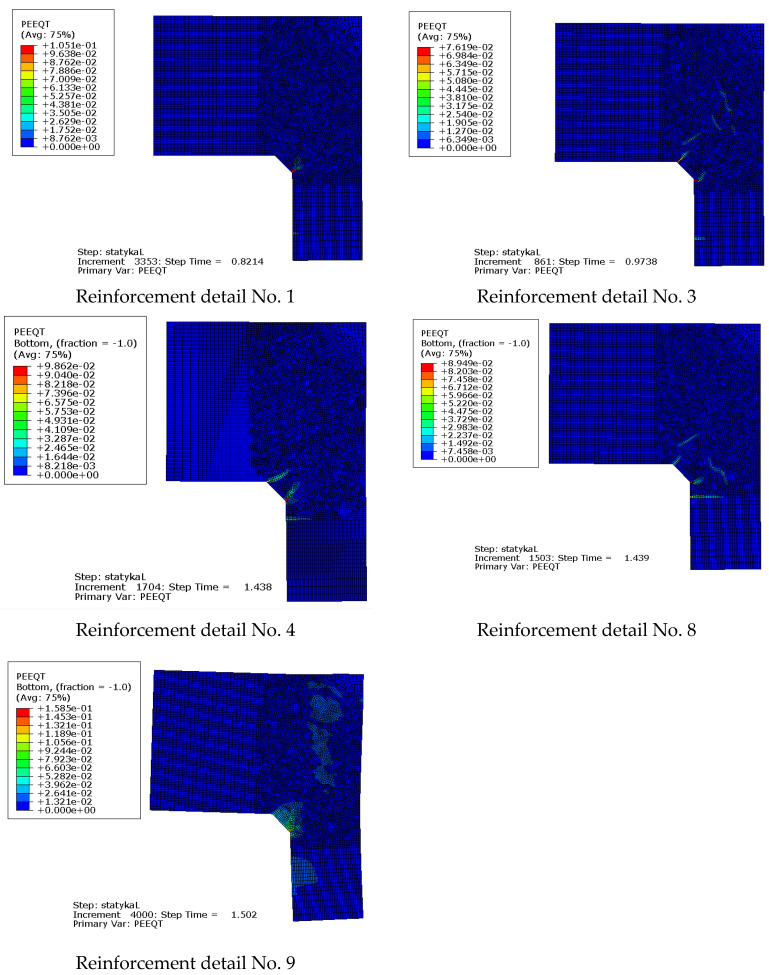
Equivalent plastic strains in tension PEEQT for the chosen reinforcement details in the plane stress state.

The equivalent plastic strains in tension PEEQT for the chosen reinforcement details in the plane stress state are listed in Figure 24. This time the propagation of cracks is seriously limited in comparison to the previous case, even for reinforcement details without diagonal stirrups. A higher cross section height of one of the components causes a completely different crack pattern and this time, crack extension is not as large as in the case of elements with the same cross section heights.

**Figure 25:**
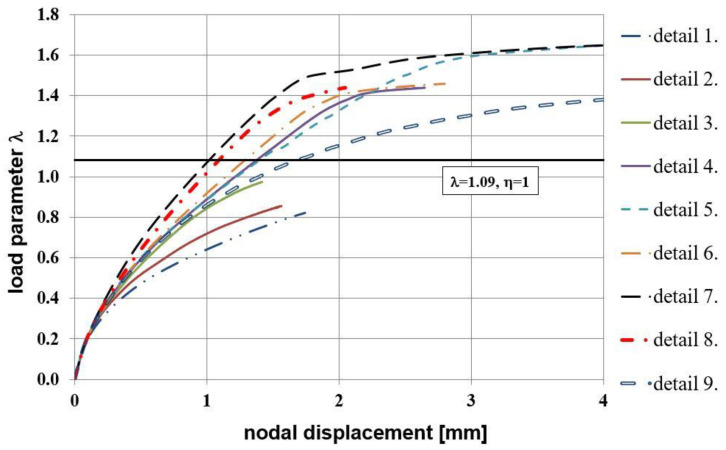
Nodal displacement vs. loading parameter in the plane stress state.

**Figure 26:**
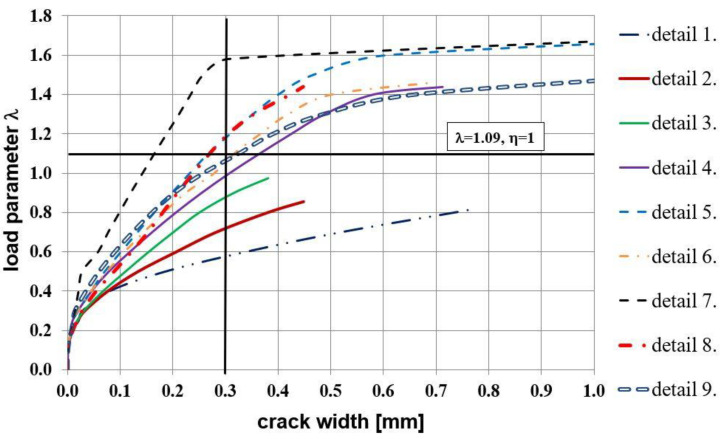
Crack width vs. loading parameter in the plane stress state.

The relationship between the nodal displacement and the loading parameter is presented in Figure 25. A worse performance of the details without diagonal stirrups (No. 1 to 3) and detail No. 9 in the plane stress state is observed. The most important results concern crack width for all the details—see Figure 26. This time, only three reinforcement details in the plane stress state reach a satisfactory loading parameter when the limit crack width of 0.3 mm is reached. Finally, the authors draw the conclusion that for the case of the different cross section heights, details No. 7 and 8 are recommended for practical use.

Values of efficiency factors are listed in Table 8. Please note that this time the factors obtained in the plane strain state seem to be too optimistic, especially for the details without diagonal stirrups. A structural designer should consider this situation very carefully as the crack width criterion is decisive in the case of elements with the different cross section heights. From all the examined reinforcement details the original detail No. 8 proposed by the authors seems to be the most recommendable of all the analyzed details.

#### 4.2.3. Influence of Reinforcement Ratio on the Efficiency Factor

**Figure 27:**
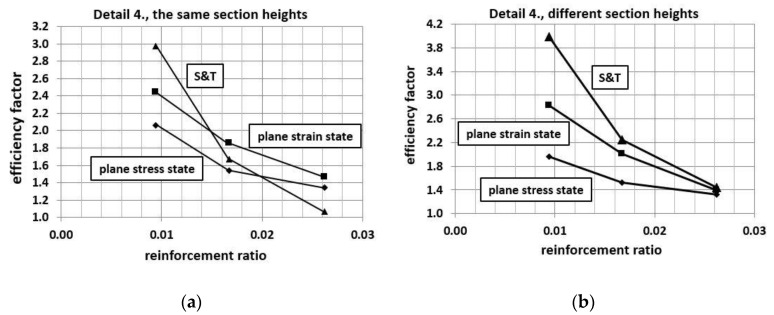
Corner efficiency factor vs. reinforcement ratio—detail No. 4, (**a**) the same section heights, (**b**) different section heights.

It is commonly known (McGregor [55], Campana et al. [56]) that the reinforcement ratio of the adjacent elements has a large influence on the efficiency factor of the corner—as the reinforcement ratio increases, the efficiency factor decreases. This trend is identical for different reinforcement details and different solutions of anchorage of the main reinforcement (loops, hooks or bends). The authors decided to check whether this trend is properly reproduced by their approaches (S&T and FEM) on the example of reinforcement detail No. 4 with the same and different cross section heights of adjacent elements. Additional computations were conducted using 2ϕ12 and then 2ϕ16 reinforcement both top and bottom. All the results confirm the trend reported in the literature—see Figure 27. The largest decrease of the efficiency factor occurs in the S&T analysis.

#### 4.2.4. Dependence of the Results on the Diagonal Reinforcement Area

As suggested in the work of Leonhardt [57] and in DIN 1045-1 [58], the corner efficiency factor strongly depends not only on the main reinforcing bars but also on diagonal bars applied. For this reason, the authors decided to perform a numerical study on the impact of the diagonal reinforcement area on the corner efficiency factor.

**Figure 28:**
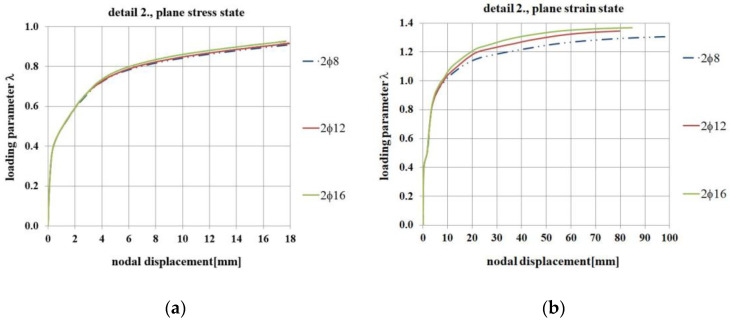
Nodal displacement vs. loading parameter for different diagonal reinforcement areas, (**a**) in the plane stress state, (**b**) in the plane strain state.

**Figure 29:**
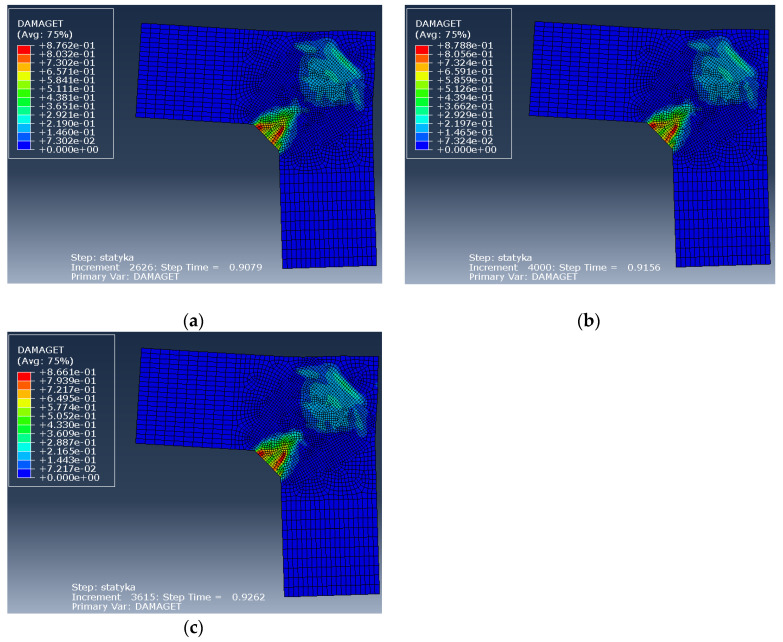
DAMAGET maps in the plane stress state for different areas of diagonal reinforcement: (**a**) 2ϕ8 mm, (**b**) 2ϕ12 mm, (**c**) 2ϕ16 mm bars.

**Figure 30:**
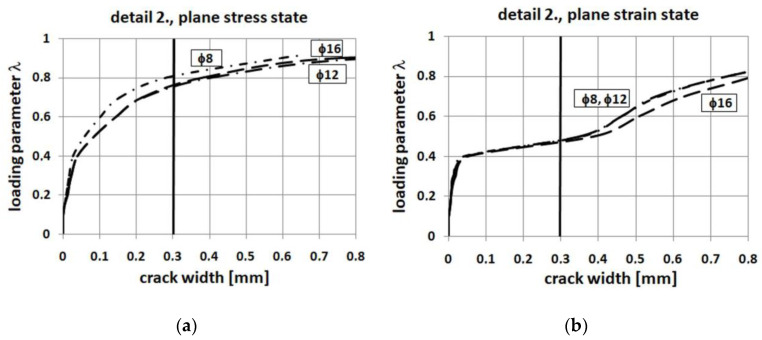
Crack width for different areas of diagonal reinforcement, (**a**) the plane stress state, (**b**) the plane strain state.

For this purpose, in Abaqus the authors analyzed reinforcement detail No. 2 (the case of elements with the same cross section heights) using three values of the diagonal reinforcement area, namely 2ϕ8 mm (initially calculated in the S&T analysis), 2ϕ12 mm and 2ϕ16 mm bars. Calculations were performed in both the plane stress and plane strain states. Selected results of the calculations, namely the nodal displacement, DAMAGET maps, and the crack width are presented in Figure 28, Figure 29 and Figure 30.

The results obtained in this parametric study show that there is no significant difference if 2ϕ8, 2ϕ12, or 2ϕ16 mm diagonal bars are used. In the plane stress state, the curves presented in Figure 28 almost coincide and in the plane strain state the maximum loading parameter *λ* is only somewhat higher when using larger areas of diagonal reinforcement. By contrast, the results presented in Figure 30 concerning crack widths are slightly in favor of a smaller area of diagonal reinforcement (which is probably caused by the better bond between concrete and reinforcement for smaller diameters).

## 5. Comparison of Numerical Results with Laboratory Tests

### 5.1. Efficiency Factors for the Case of Elements with the Same Cross Section Heights

A few other researchers mentioned in Section 1 performed laboratory tests of corners under opening bending moment. All these tests only considered the case of elements with the same cross section heights. Although the analyzed corners were made of different materials and had different dimensions, the corner efficiency factor can serve as an objective criterion for the comparison of all the tests with numerical results presented in the paper. All the efficiency factors obtained in the paper are compared with the results of the laboratory tests for the case of elements with the same cross section heights in Table 9. For reinforcement details No. 1 and 2, the experimental efficiency factors are even lower than in S&T and FEM computations. Surprisingly details No. 4 and 5 did not reach the efficiency factor of *η* = 1 in the laboratory tests. Finally, details No. 6 and 7 show the highest efficiency factors in all the tests. Both these details can be recommended as rational, but it is easier to make detail No. 7 on the construction site. The comparison is approximate and indicative because of different dimensions, material properties and reinforcement ratios adopted by different authors.

### 5.2. Comparison with Johansson’s Laboratory Tests

Johansson [13] performed laboratory tests on RC frame corners under closing and opening bending moment. The authors numerically recreated a laboratory test of the specimen marked RV9 in Johansson’s research. A displacement control was applied with a displacement rate from 0.15 to 0.30 mm/min. The reinforcement in the laboratory test was as follows: looped main bars and a diagonal bar, which makes this reinforcement detail similar to reinforcement detail No. 2 presented in Table 2. The only difference is that in the authors’ research, the main bars were not loop-shaped.

The RV9 specimen was modeled in Abaqus software using the CDP model for concrete and the classical metal plasticity for reinforcing steel. The material properties were assumed as identical to those in Johansson’s research:

- Concrete: *f_c_* = 32.2 MPa, *E_c_* = 31 GPa, *ν* = 0.2, *f_t_* = 2.6 MPa, *G_f_* = 136.4 N/m,
- Reinforcing steel: *f_y_* = 570 MPa, *E_s_* = 200 GPa, *ν* = 0.3.

The dilatation angle was assumed equal to 15 degrees and the relaxation time—equal to 0.0001 s.

**Figure 31:**
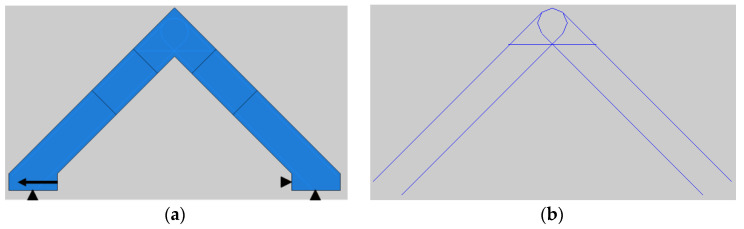
Boundary conditions (**a**) and reinforcement (**b**) of modeled RV9 specimen.

**Figure 32:**
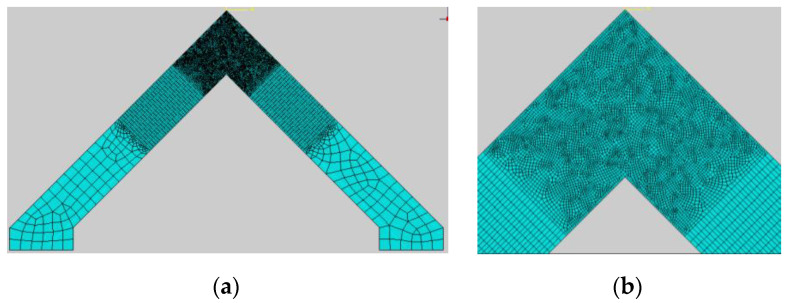
Meshing of modeled RV9 specimen (**a**) with zoomed corner zone (**b**).

A general view of the specimen, the boundary conditions applied in Abaqus and the reinforcing bars are presented in Figure 31; the yellow arrow represents the nodal displacement, which is imposed in an identical manner as was the case in the laboratory test. The specimen RV9 was meshed mainly using quadrilateral finite elements (the so-called “quad-dominated” element shape control in Abaqus) with FE sizes in the range of 75 to 5 mm—see Figure 32.

The main reinforcement of the adjacent members was 5ϕ16 bars with additional diagonal bars 3ϕ16. The full bond between steel and concrete was assumed (the “embedded region” option in Abaqus code). Computations were performed in the plane stress state using the full-Newton solution technique. The parameters of the CDP model were assumed as identical to those in the previous section—see Table 6. The authors decided to present the results of the force–displacement relationship and PEEQT maps (propagation of cracks) obtained in FEM calculations and compare them with the laboratory test results.

**Figure 33:**
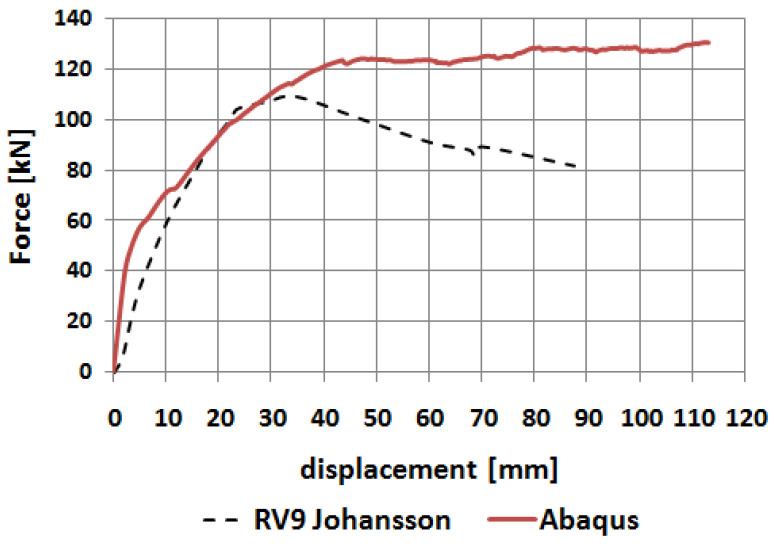
Force–displacement relationships obtained in FEM analysis and laboratory tests.

The relationship between the horizontal reaction force and the imposed displacement is presented in Figure 33 and compared with the same relationship obtained in Johansson’s laboratory test (dashed line). Both curves appear to be similar whereas the curve obtained in Abaqus has no peak and no descending part. Instead of this it has a clear plateau similar to that presented in Figure 21. Moreover, the curve obtained in the FEM shows a higher stiffness at the beginning of the loading process. Despite these shortcomings it can be concluded that both curves are comparable, and the FEM analysis properly reflects the laboratory test.

**Figure 34:**
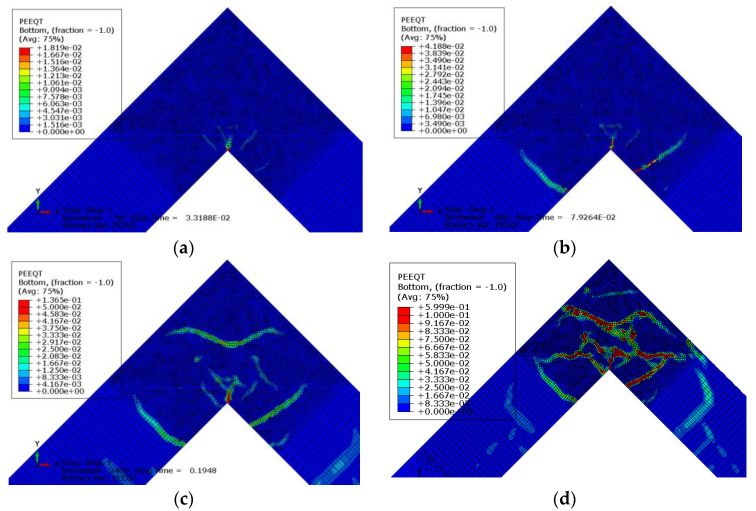
(**a**–**d**) PEEQT maps in FEM analysis in four selected time steps.

**Figure 35:**
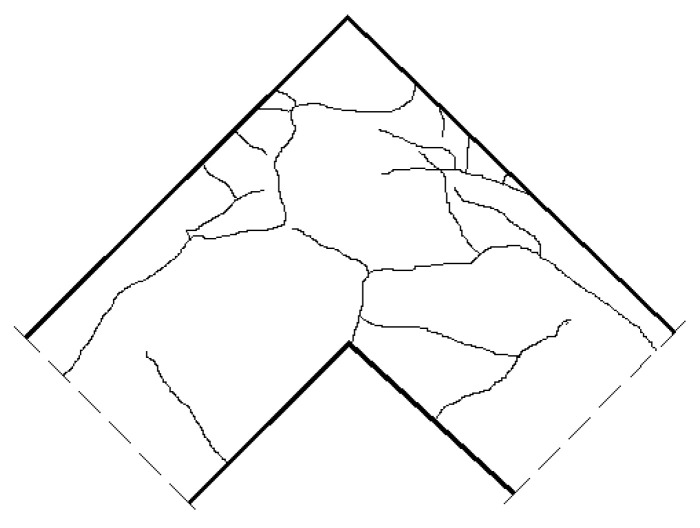
A sketch of the crack pattern obtained in Johansson’s laboratory tests (reprinted with permission from Johansson [13], Copyright 2000, M. Johansson).

A crack pattern obtained in the FEM analysis is presented in Figure 34 for four selected time steps, where the time value *t* = 1 s is equivalent to the maximum displacement in the displacement-control procedure (120 mm). The first figure presents a map of PEEQT at the very beginning of the loading process. Despite not being particularly large, a clear crack can be seen propagating from the concave angle of the corner zone. Using formula (18) the authors calculated a maximum crack width with a value of *w* = 0.25 mm. Afterwards two new concurrent cracks appear further from the concave angle as shown in the second map of PEEQT. At this step time, the maximum crack width is *w* = 0.57 mm which is a very large value in comparison to the typical values of ultimate crack widths given by codes and handbooks. The excessive value of the crack width is in accordance with findings from the previous section that a reinforcement detail containing only one diagonal bar is not recommended for a corner under opening bending moment. In the third map, a new crack propagating in the corner zone where the reinforcement is loop-shaped is apparent. The current crack width is *w* = 1.84 mm. The fourth figure presents a PEEQT map from the end of the FEM computations and some new cracks and extensions of the previous cracks appear. Eventually the crack width reaches *w* = 8.10 mm. The final crack pattern can be compared with the crack pattern obtained in Johansson’s laboratory test—see Figure 35. There are similarities between the FEM and laboratory results—firstly, the comparable crack propagation from the concave corner and cracks outside the corner zone perpendicular to the axes of adjacent elements. Secondly, there is also a very similar extension of the crack propagating from the concave corner. However, there is one clear difference—there are cracks located along the loop-shaped fragments of the reinforcing bars and none of them appears in the FEM analysis. Despite this, a fairly strong agreement between the FEM simulations and laboratory test is observed and therefore the CDP model with the properly calibrated parameters can be recommended for practical use.

## 6. Conclusions

A combination of S&T and FEM computations was carried out to find the most rational reinforcement detail for frame corners under opening bending moment. The following conclusions can be drawn:

(1) It is possible to choose a rational reinforcement detail for a corner under opening moment even in the case of elements with different cross section heights using a combination of the S&T method and FEM.
(2) The most recommendable details are detail No. 7 in the case of elements with the same cross section heights (see Table 2) and details No. 7 and 8 in the case of elements with different cross section heights (see Table 2).
(3) There are no significant differences in the obtained results when applying the different areas of the diagonal bars (2ϕ8, 2ϕ12 or 2ϕ16 mm).
(4) The use of diagonal bars only is insufficient to obtain a satisfactory efficiency factor and a limited crack width; these goals can only be achieved by using diagonal stirrups.
(5) The reinforcement ratio of the adjacent elements has a large influence on the efficiency factor of the corner, namely an increase in the reinforcement ratio causes a decrease in the efficiency factor.
(6) It is possible to assume simpler and still appropriate S&T truss schemes than that used in Eurocode 2 [1] and handbooks for a corner under opening bending moment, even in the case of the use of corner elements with different cross section heights.
(7) When using the CDP model its parameters should be assumed in a careful way. The authors recommend calibrating and validating some of these parameters. To obtain realistic results, the authors propose the following values of CDP parameters (see the discussion in Section 3):
  - A relaxation time of 0.0001 s or less (for the loading time 1 s);
  - A dilatation angle of 5 to 15 degrees.

## Figures and Tables

**Table 1:** Corner efficiency factors obtained in the laboratory tests of various authors.

Reinforcement Detail	Efficiency Factor	Reinforcement Detail	Efficiency Factor
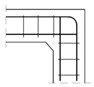	0.43 (Mayfield et al. [4]) 0.66 (Kordina and Wiedemann [7]) 0.46 (Al-Khafaji et al. [11])	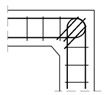	0.78 (Mayfield et al. [4])
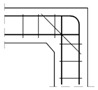	0.59 (Mayfield et al. [4]) 0.55 (Skettrup et al. [9])	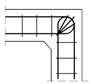	1.32 (Kordina and Wiedemann [7]) 1.13 (Skettrup et al. [9])
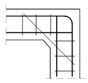	1.07 (Moretti and Tassios [12])	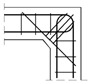	1.12 (Moretti and Tassios [12])
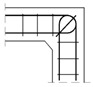	0.88 (Mayfield et al. [4])		

**Table 2:** Reinforcement details analyzed in the research.

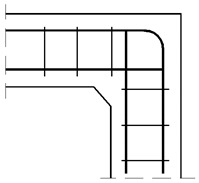	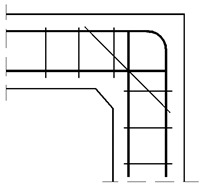	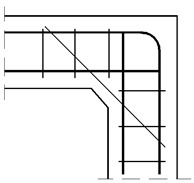
Detail No. 1	Detail No. 2	Detail No. 3
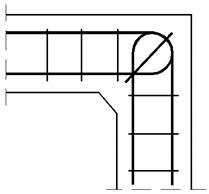	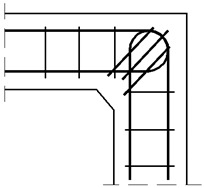	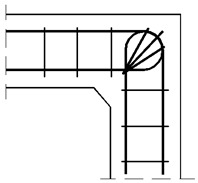
Detail No. 4	Detail No. 5	Detail No. 6
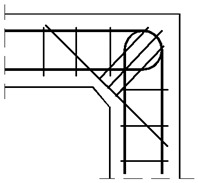	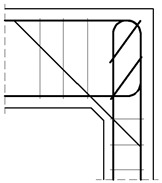	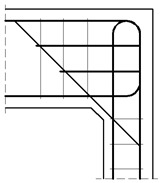
Detail No. 7	Detail No. 8	Detail No. 9

**Table 3:** Values of dilatation angle as suggested by other authors.

Source	Dilatation Angle *ψ* [°]
Jankowiak [43]	49
Genikomsou and Polak [40]	38
Mostafiz et al. [44]	38
Kmiecik and Kamiński [45]	36
Malm [46]	25–38
Menetrey [47]	10
Mostofinejad and Saadatmand [48]	0
Marzec [49]	8 or 10
Rodriguez et al. [50]	30
Urbański and Łabuda [51]	15

**Table 4:** Efficiency factors and provided reinforcement for each reinforcement detail—the case of elements with the same cross section heights (maximal value in bold).

Detail No.	Efficiency Factor Obtained in S&T	Decisive Element (No. of Strut or Node)	Provided Reinforcement, Diameters Given in (mm)
1.	0.70	Strut No. 5	main reinforcement: 2ϕ20 top and 2ϕ20 bottom, no loops
2.	0.64	Strut No. 9	2 diagonal bars ϕ8 each, no loops
3.	0.66	Strut No. 5	2 diagonal bars ϕ8 each, no loops
4.	0.94	Strut No. 5	diagonal stirrup ϕ16, looped main bars
5.	1.21	Nodes N1, N2, N4, N5	central diagonal stirrup ϕ16, outside stirrups ϕ10, looped main bars
6.	1.25	Nodes N2, N3, N4, N5	central diagonal stirrup ϕ16, outside stirrups ϕ12, looped main bars
7.	**1.31**	Struts No. 14 and 15	2 diagonal bars ϕ16 each, central diagonal stirrup ϕ12, outside stirrups ϕ16, looped main bars

**Table 5:** Efficiency factors and provided reinforcement for each reinforcement detail—the case of elements with different cross section heights (maximal value in bold).

Detail No.	Efficiency Factor Obtained in S&T	Decisive Element (No. of Strut or Node)	Provided Reinforcement, Diameters Given in (mm)
1.	0.61	Strut No. 5	main reinforcement: 2ϕ20 top and 2ϕ20 bottom in a smaller cross section (column), and 2ϕ12 top and 2ϕ12 bottom in a larger cross section (beam), no loops
2.	0.58	Strut No. 5	2 diagonal bars ϕ8 each, no loops
3.	0.60	Strut No. 5	2 diagonal bars ϕ8 each, no loops
4.	**1.44**	Nodes N1, N2, N3	diagonal stirrup ϕ16, looped main bars
5.	1.23	Nodes N1, N2, N5	diagonal stirrups ϕ12, looped main bars
6.	1.15	Nodes N2 and N3	diagonal stirrups ϕ12, looped main bars
7.	1.17	Node N7	2 diagonal bars ϕ16 each, central diagonal stirrup ϕ16, outside stirrups ϕ12, looped main bars
8.	1.07	Nodes N3 and N4	diagonal bar ϕ8, diagonal stirrups ϕ12, looped main bars
9.	0.88	Nodes N1 and N2	2 horizontal stirrups ϕ8 each, diagonal bar ϕ8, looped main bars

**Table 6:** CDP model parameters used in FEM calculations.

Dilatation Angle *ψ* (degree)	Flow Potential Eccentricity *e*	Ratio *f_b_*_0_*/f_c_*_0_	Ratio *K*	Viscosity Parameter *μ* (s)	Fracture Energy *G_f_* (N/m)
15	0.1	1.16	0.667	0.0001	146.5

**Table 7:** Efficiency factors for all reinforcement details obtained using all methods (maximal values in bold).

Detail No.	Efficiency Factor S&T	Efficiency Factor FEM, Plane Stress	Efficiency Factor FEM, Plane Strain
1.	0.70	0.75	1.01
2.	0.64	0.79	1.11
3.	0.66	0.82	1.17
4.	0.94	1.23	1.26
5.	1.21	1.24	1.29
6.	1.25	**1.27**	1.29
7.	**1.31**	1.23	**1.32**

**Table 8:** Efficiency factors for all reinforcement details obtained using all methods (maximal values in bold).

Detail No.	Efficiency Factor S&T	Efficiency Factor FEM, Plane Stress	Efficiency Factor FEM, Plane Strain
1.	0.61	0.75	1.31
2.	0.58	0.78	1.36
3.	0.60	0.89	1.43
4.	**1.44**	1.32	1.40
5.	1.23	**1.57**	1.40
6.	1.15	1.34	1.38
7.	1.17	1.54	1.51
8.	1.07	1.32	**1.52**
9.	0.88	1.38	1.44

**Table 9:** Efficiency factors obtained in authors’ analyses and in laboratory tests—the case of elements with the same cross section heights (maximal values in bold).

Detail No.	Efficiency Factor in Laboratory Tests	Efficiency Factor S&T	Efficiency Factor FEM, Plane Stress	Efficiency Factor FEM, Plane Strain
1.	0.43 (Mayfield et al. [4]) 0.66 (Kordina and Wiedemann [7]) 0.46 (Al-Khafaji et al. [11])	0.70	0.75	1.01
2.	0.59 (Mayfield et al. [4]) 0.55 (Skettrup et al. [9])	0.64	0.79	1.11
3.	1.07 (Moretti and Tassios [12])	0.66	0.82	1.17
4.	0.88 (Mayfield et al. [4])	0.94	1.23	1.26
5.	0.78 (Mayfield et al. [4])	1.21	1.24	1.29
6.	**1.32** (Kordina and Wiedemann [7]) 1.13 (Skettrup et al. [9])	1.25	**1.27**	1.29
7.	1.12 (Moretti and Tassios [12])	**1.31**	1.23	**1.32**

## Data Availability

Not applicable.
